# Supplementary material for: X-ray nanotomography of coccolithophores reveals that coccolith mass and segment number correlate with grid size
Source: Nat Commun. 2019 Feb 14;10:751. doi: 10.1038/s41467-019-08635-x (PMC6375944; doi:10.1038/s41467-019-08635-x)
Supplement: Supplementary file 1 — Supplementary Information [file 41467_2019_8635_MOESM1_ESM.pdf]

Supplementary information

X-ray nanotomography of coccolithophores reveals that coccolith mass and segment number correlate with grid size

Beuvier et al.

Supplementary Note 1

Uncertainty of the determination of the volume and the mass of coccoliths from 3D-CXDI.

The mass of the coccoliths can be obtained assuming first that the density of coccoliths is the same as the one of calcite (2.71 g/cm^3) and secondly that the volume of the coccoliths is correctly determined. This last point is not so trivial in particular because the voxel size of the real matrices (~30×30×30 nm^3) is not negligible compared to the size of the crystals. The coccoliths, and especially the smallest ones, have delicate shields and fine central grids. So, the determination of the volume of the coccoliths depends both on the resolution of 3D-CXDI and on the dimensions of the different parts of the coccoliths. To illustrate this point, the voxel number of three 3D matrices is plotted as a function of the voxel intensity I (data coded in 8 bits (2^8) spans from 0 to 255) for three coccoliths having different calcification degrees (**Supplementary Figure** 4). *R. parvula* is the least calcified species. Its distribution function is quite flat (purple curve). *E. huxleyi RCC1216* is more calcified than *R. parvula* (i.e. its volume is higher) and its function distribution displays a broad peak centers at around I = 205. *G. muellerae* is even more calcified and its distribution function is characterized by a finer peak. Thus it appears that the thinner and smaller the coccoliths, the broader the distribution. To estimate the volume and the associated uncertainty, one reliable method consists in plotting several 3D views having different voxel intensity values I and in determining the range of intensities for which the 3D views have acceptable shape. This is highlighted in the inset of Supplementary Figure 4 for the most complicated species, i.e. *R. parvula*. In this example, the calcification of the coccolith is clearly overestimated for $I<20$ (in red). This can be seen by looking either at the 3D views or at the out-of-plane cross sections. For intermediate values, i.e. $20<I<40$ (in green), the 3D morphology/shape is in agreement with SEM analysis. And for $I>40$ (in blue), it is clear that the coccolith is not calcified enough compared to SEM images,

i.e. the volume and mass are thus underestimated with these voxel intensities. These results show that the mass uncertainty comes mainly from the difficulty to determine accurately the interface between air and calcite. This can be translated as "The smaller the specific surface of a coccolith, the higher the relative mass uncertainty". For the 45 coccoliths that are segmented in this study, the specific surface areas, calculated from the Chimera software, are ranging from S = 4.4±0.2 m^2/g for the heavier coccolith of *G. oceanica*, to 12.8±2.9 m^2/g for *R. parvula*.

Supplementary Figures

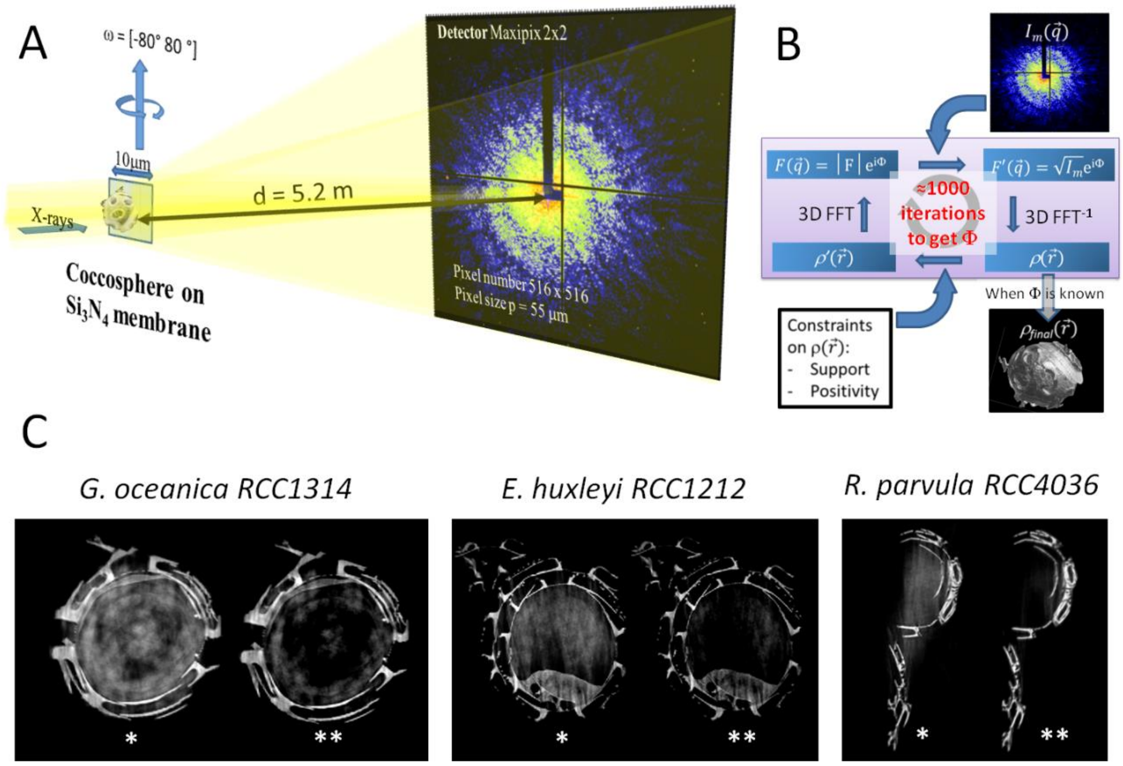

Supplementary Figure 1. Principle of 3D-CXDI and flattening. (**A**) A plane wave illuminates a coccosphere, and an oversampled diffraction pattern is measured by a detector for ω = -80°(±5°) to +80°(±5°) to obtain the 3D view of the intensity in the reciprocal space. (**B**) Phase retrieval algorithms iterate back and forth between 3D real and 3D reciprocal spaces. In each iteration, various constraints, including support, positivity (i.e. electron density cannot be negative), or partially overlapping regions, are enforced in real space, while the measured Fourier magnitudes are imposed in the updated reciprocal space. (**C**) 2D slices of 3 coccolithophores (*) without and (**) with the flattening correction.

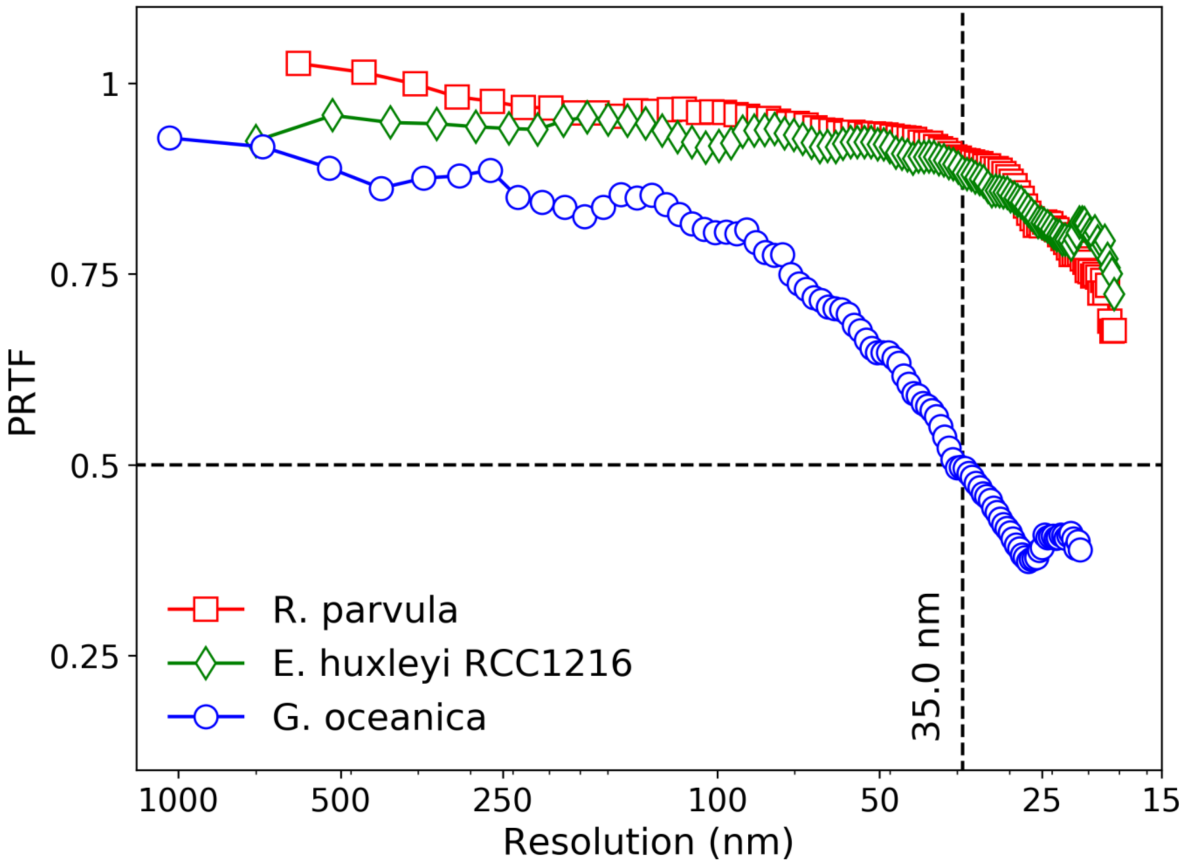

Supplementary Figure 2. PRTFs and real resolutions of CXDI measurements. Phase retrieval transfer function of the final images obtained for the coccolithophore *R. parvula*, *E. huxleyi RCC1216* and *G. oceanica* as a function of the resolution. The voxel size is 28.8×28.8×28.8 nm^3 for *R. parvula* and *E. huxleyi RCC1216*. At a resolution of 28.8 nm, the PRTFs are higher than 0.5. The real resolutions are thus close to the voxel size for the experiment done on these 2 species. For *G. oceanica*, the voxel size is 32.5×32.5×32.5 nm^3. For PRTF = 0.5, the real resolution is equal to 35.0 nm. This value is thus slightly higher than the voxel size.

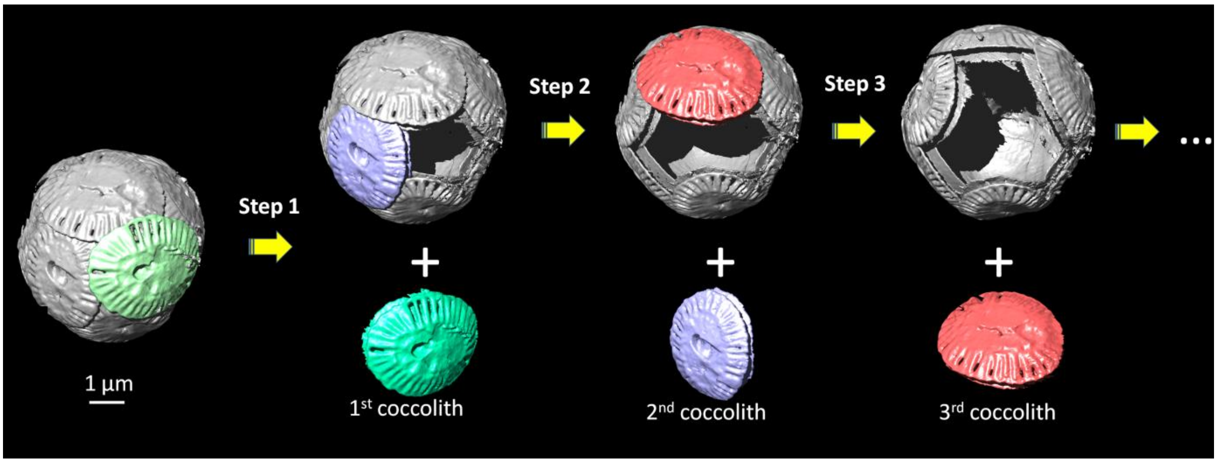

Supplementary Figure 3. Segmentation procedure. Scheme showing the methodology used to extract the coccoliths from the coccosphere (*E. huxleyi P41*). During the step 1, the whole coccosphere containing C_N = 14 coccoliths is segmented in two parts: a first coccolith and a new coccosphere containing now 13 coccoliths. This procedure is repeated during the following steps. After 14 cycles of segmentation, the 14 coccoliths are obtained.

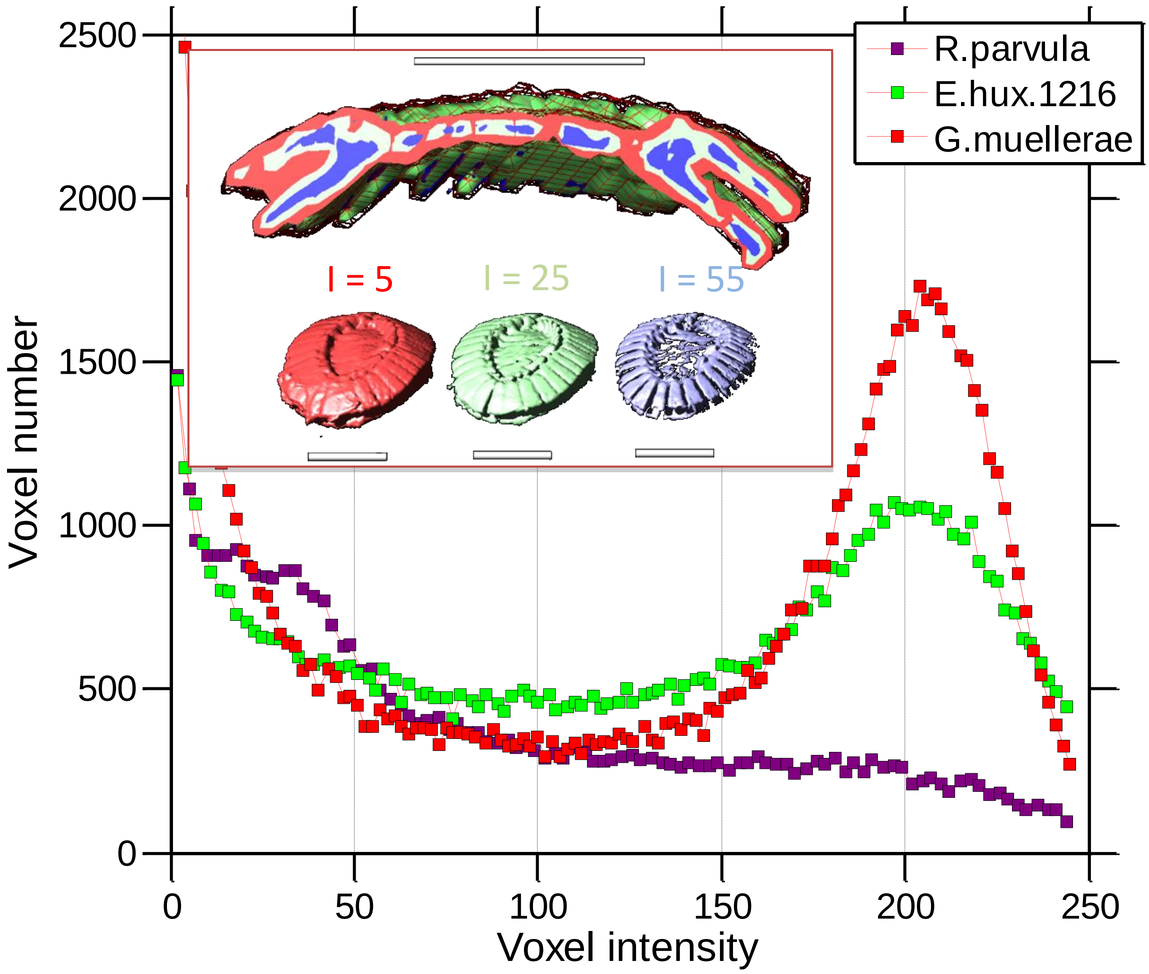

Supplementary Figure 4. Uncertainty of the mass determination by 3D-CXDI. Voxel number as a function of the voxel intensity *I* for three matrices of three coccoliths with voxel size 28.8×28.8×28.8 nm^3. Amongst the three species, *R. parvula* is the lowest calcified species, *G. muellerae* the highest. In inset, an out-of-plane cross section of the coccolith of *R. parvula* is shown for three voxel intensity values *I*. For *I = 5* (shown in red), the volume is overestimated V = 0.88 μm^3. For *I = 25* (shown in green), the volume is correctly estimated V = 0.48 μm^3. For *I = 55* (shown in blue), the volume is underestimated V = 0.19 μm^3. For this coccolith, the acceptable *I* values are ranging from 20 and 40. This corresponds to volumes ranging from 0.32 to 0.55 μm^3 and to masses from 0.88 to 1.50 pg. Scale bar = 1 µm.

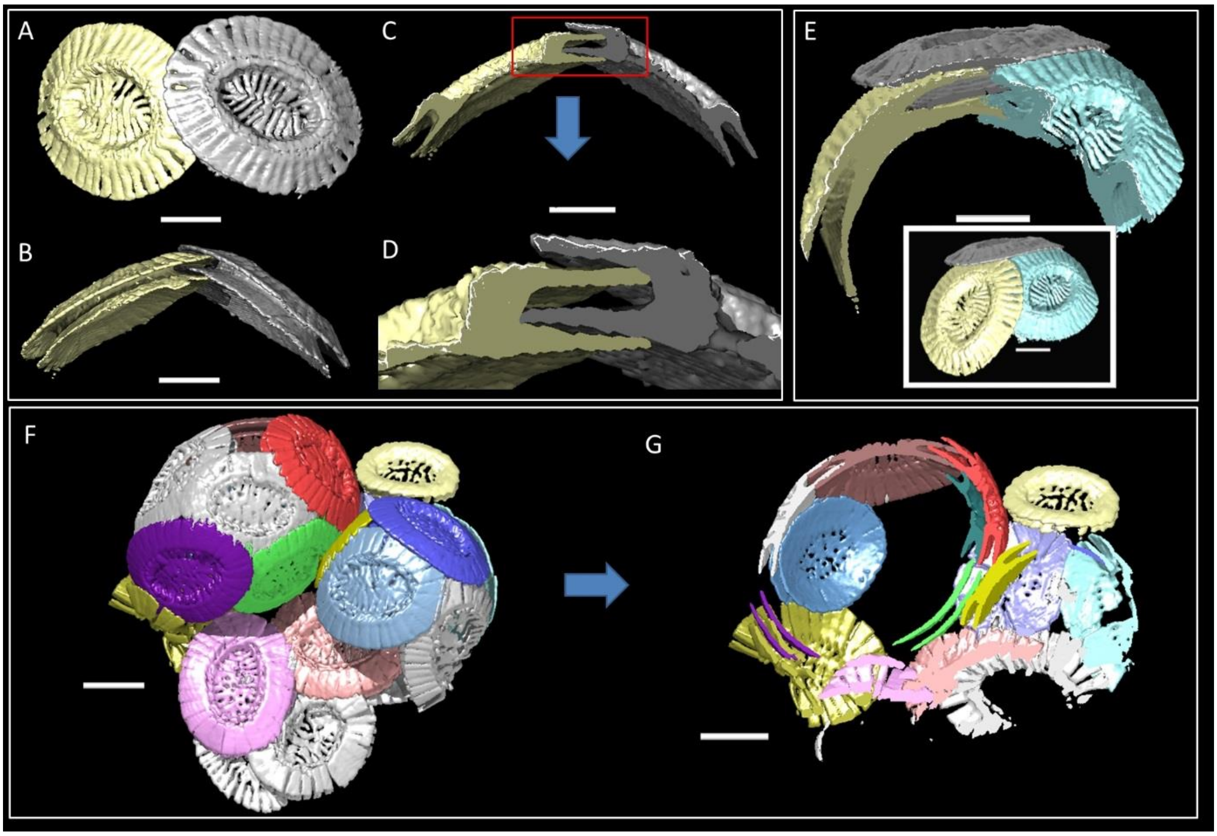

Supplementary Figure 5. Uncertainty of the segmentation due to the tight imbrications between the coccoliths. Another error on the mass determination comes from the difficulty for some coccospheres to segment the coccoliths due to the tight imbrications between them. (**A** to **E**) 3D-CXDI of coccoliths extracted from the coccosphere of *E. huxleyi RCC1216* showing the tight imbrications between them. (**A**) Distal view. (**B**) Side view. (**C**) Side view after cutting half of each coccolith. (**D**) Zoom of C. (**E**) 3D-CXDI of three coccoliths.(**F**) 3D-CXDI of the coccosphere of *R. parvula* after colorizing the coccoliths. (**G**) Half of the coccosphere was cut to highlight the tight imbrication between the coccoliths. Scale bar = 1µm.

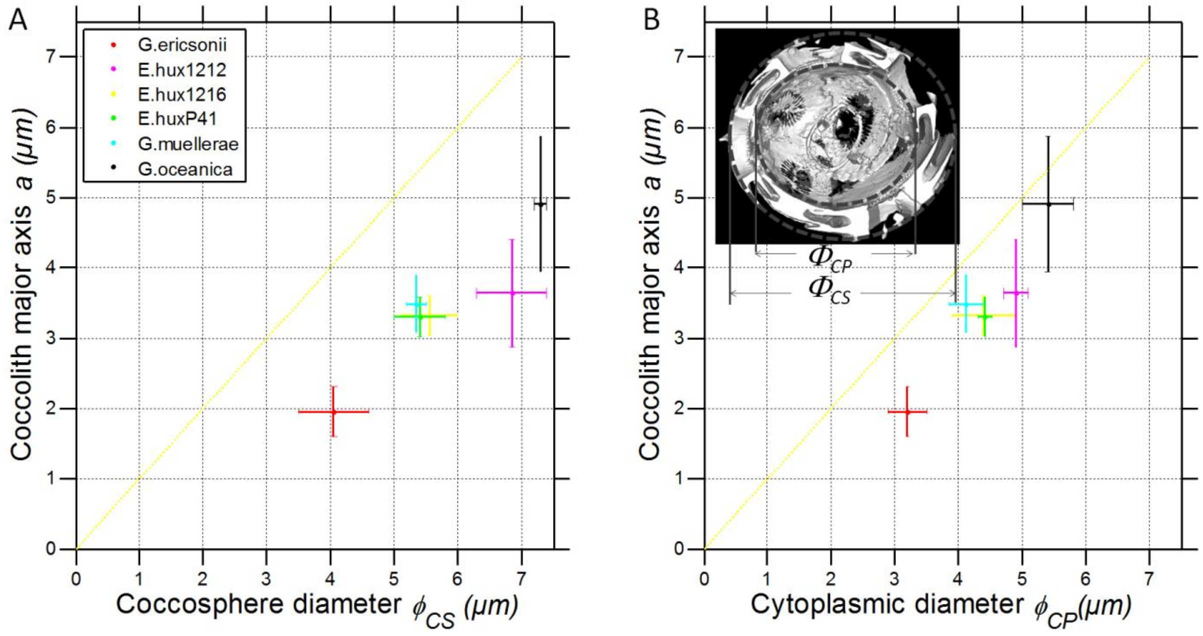

Supplementary Figure 6. Coccoliths major axis *a* as a function of the coccolithophore size. Even though the size variability of the coccoliths within a coccosphere is high, there is a positive correlation between the size of coccoliths (i.e. the coccolith major axis *a*) and the size of the coccolithophores. This can be shown either by looking at the diameter of the coccospheres or the one of the cytoplasmic cells. (**A**) Coccolith major axis *a* as a function of the coccosphere diameter Φ_{CS}. (**B**) Coccolith major axis *a* as a function of the cytoplasmic diameter Φ_{CP}. The error bar on the x-axis originates from the flattening of the coccolithophore. The y-axis error bar represents the major axis variability of coccoliths within a coccosphere. In inset of (**B**), half of a coccolithophore *G. oceanica* is plotted to illustrate the meaning of Φ_{CS} and Φ_{CP}. For *R. parvula*, Φ_{CS} and Φ_{CP} are not determined as the coccosphere was broken.

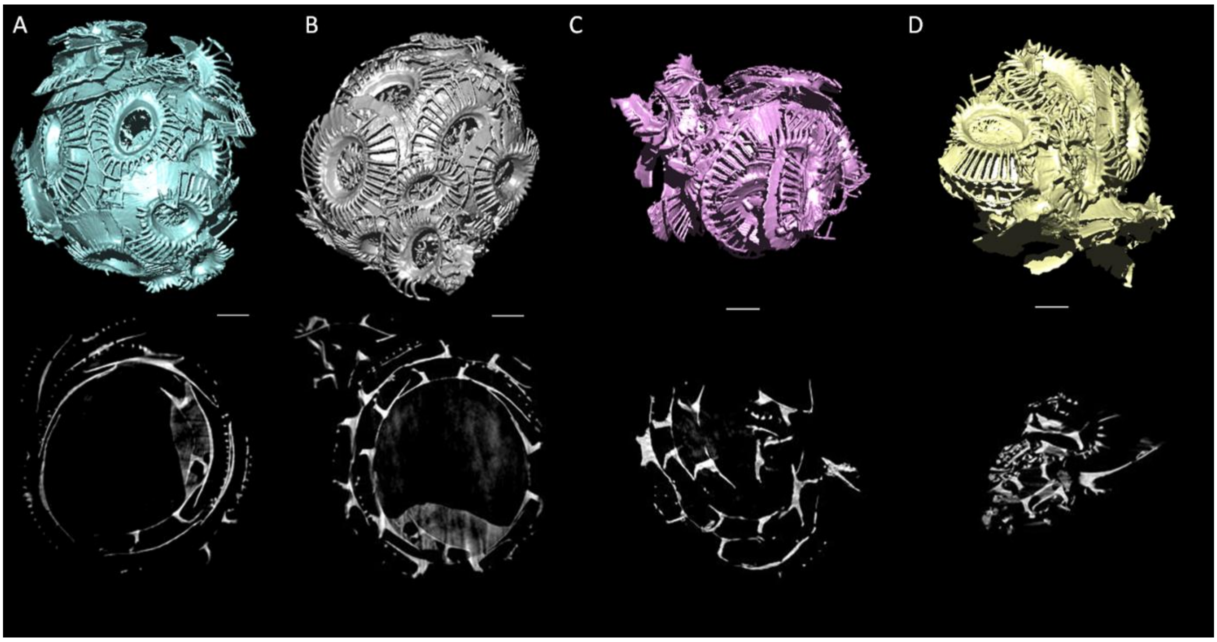

Supplementary Figure 7. Four different coccospheres of *E. huxleyi RCC1212* observed by 3D-CXDI. (**A** and **B**) 3D-CXDI of two almost spherical coccospheres with (top) 3D views and (bottom) slices. (**C** and **D**) 3D-CXDI of collapsed coccospheres with (top) 3D views and (bottom) slices. Scale bar = 1 µm. On the contrary to *R. parvula* (see **Supplementary Figure** 10), it was not possible to find isolated single segments.

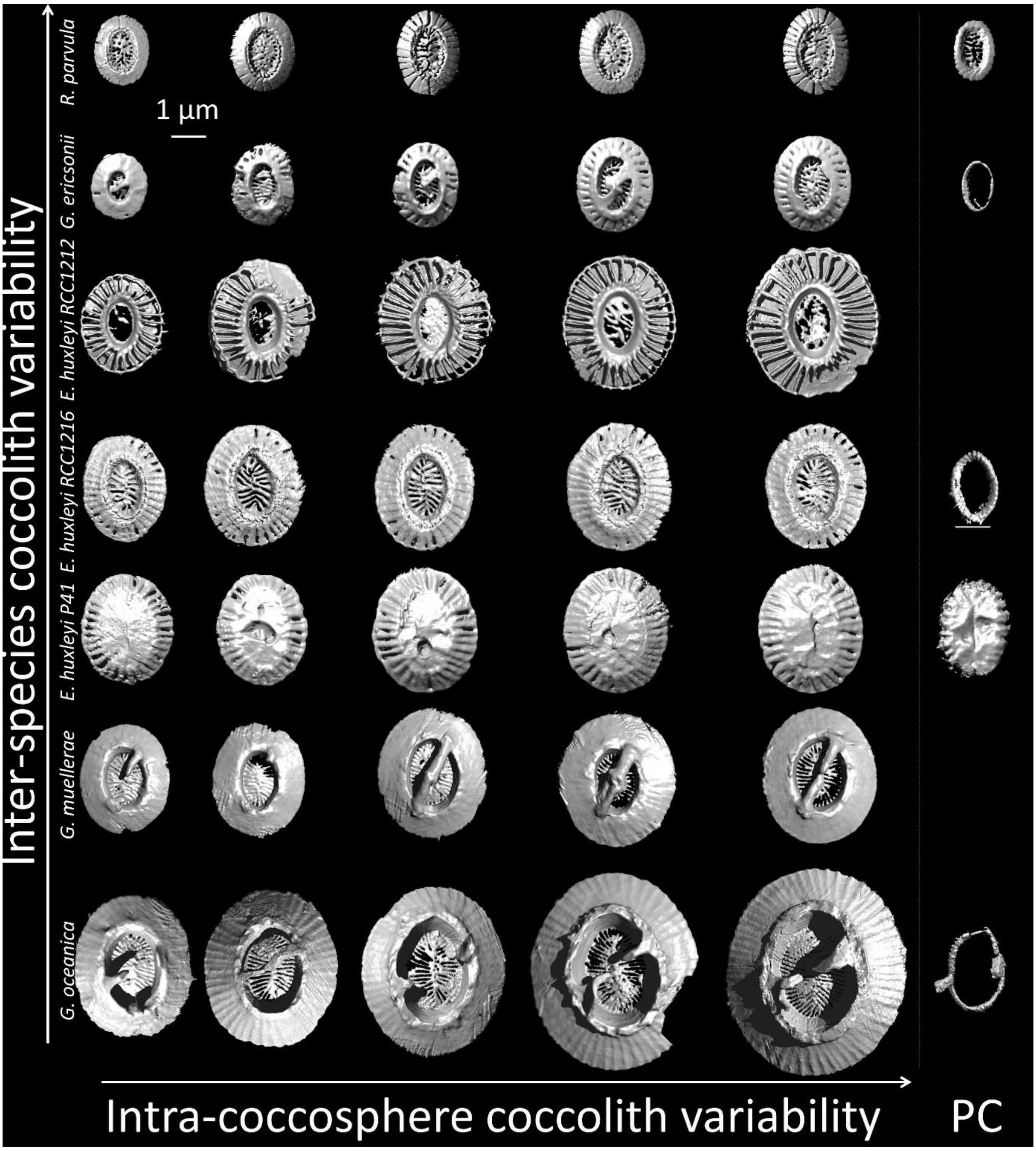

Supplementary Figure 8. Size variability of the coccoliths and protococcoliths observed in distal view. On each line, 5 coccoliths were extracted from the same coccosphere. Line 1: *R. parvula*; line 2: *G. ericsonii*; line 3: *E. huxleyi RCC1212*; line 4: *E. huxleyi RCC1216*; line 5: *E. huxleyi P41*; line 6: *G. muellerae*; line 7: *G. oceanica*. The right column displays the protococcoliths *pc* extracted inside the coccospheres. This figure highlights the size coccolith variability within a coccosphere (i.e. the intra-coccosphere coccolith variability) and between

species (i.e. the inter-species coccolith variability). In addition, the right column displays the protococcoliths *pc* extracted from the core of the coccospheres. This observation confirms that the formation of a coccolith begins with the nucleation and growth of a ring (i.e. the protococcolith ring). Scale bar = 1 µm.

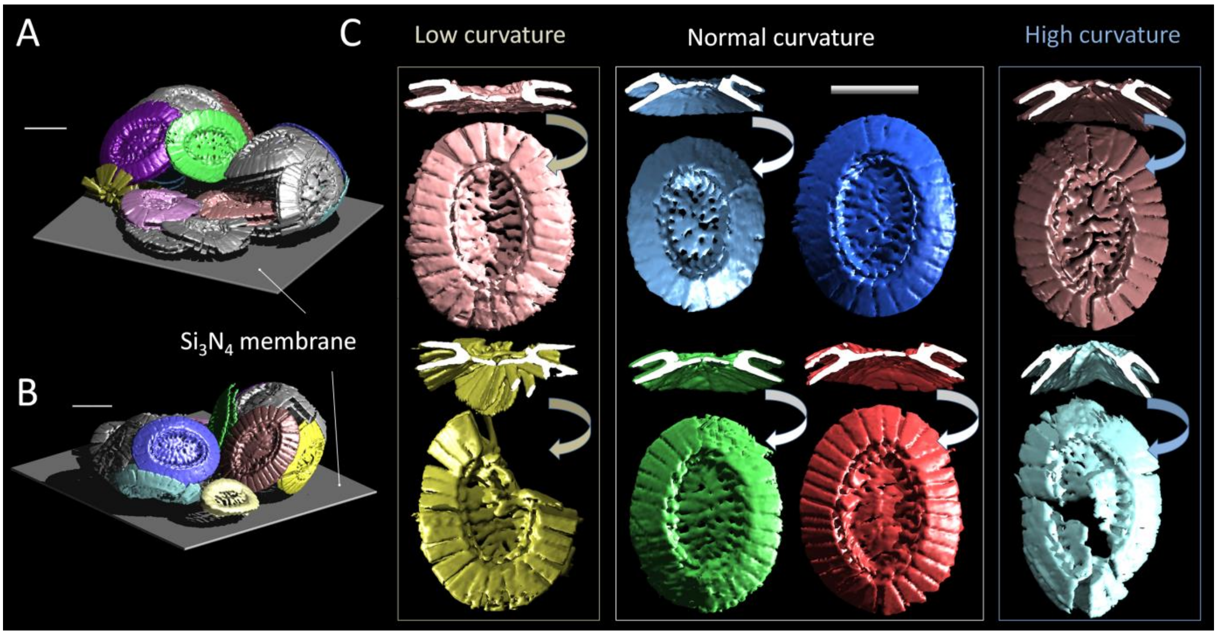

Supplementary Figure 9. Deformation of some coccoliths due to the mechanical constraints with the Si_3N_4 membrane in the case of *R. parvula*. (**A** and **B**) 3D-CXDI of the coccospheres with two different views. The Si_3N_4 membrane was artificially added to understand the origin of the deformation of some coccoliths. (**C**) Coccoliths segmented from the coccospheres shown in A and B. Some coccoliths are deformed and exhibit a low out-of-plane curvature/inclination (left panel). Some coccoliths are intact and have a normal curvature (middle panel), i.e. the inclination of the shields is typically by about α~30±5° along the major axis and α~25±5° along the minor axis for normal coccoliths. Others are deformed in such a way that the out-of-plane inclination is higher. The inclination of the shields can be thus overestimated for flattened coccoliths (exhibiting low curvature) and underestimated for bended coccoliths (high curvature).

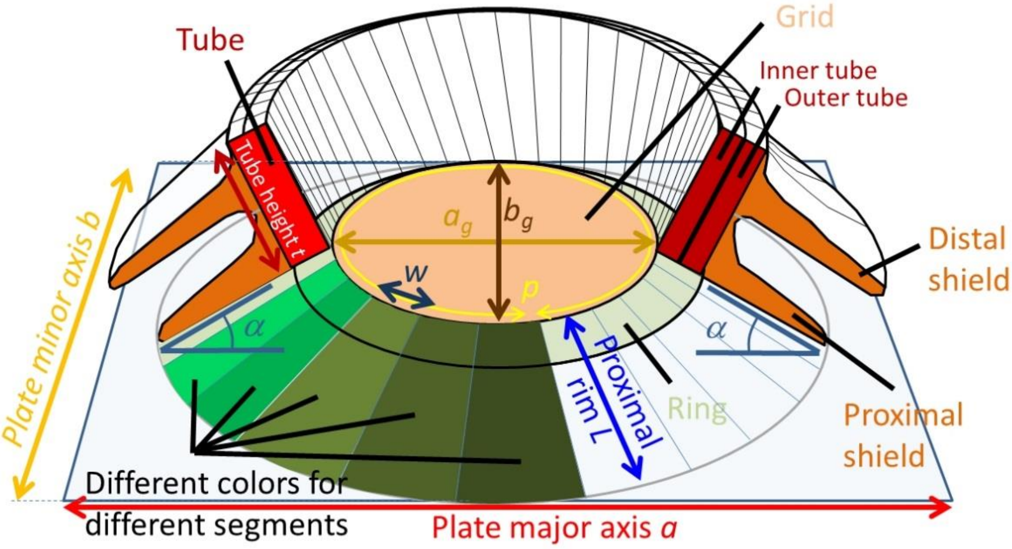

Supplementary Figure 10. Sketch of a coccolith. The parameters of this study *a*, *b*, α, *p*, *L*, *t* and *w* are schematized.

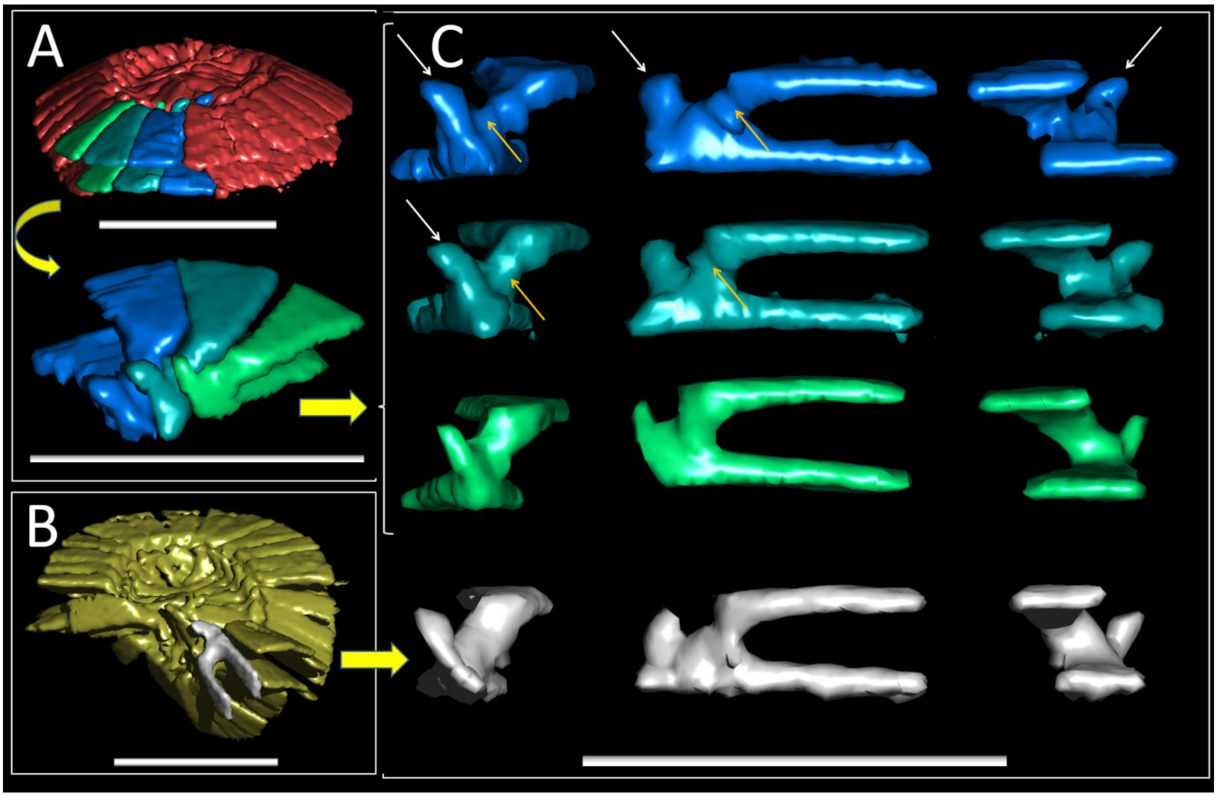

Supplementary Figure 11. 3D geometry of the segments of *R. parvula* resulting from 3D-CXDI measurements and the segmentation step. (**A, B**) Coccoliths extracted from the coccosphere of the *R. parvula* species. In A, 3 segments were extracted and drawn in different colors. In B, one segment was extracted. (**C**) The extracted segments were individually plotted. The inner part of the tube (shown by white arrows) looks like a tip of around 100 nm height built-in to the neighbor tube segment. This architecture may strengthen the interlocking between the R-unit segments. The orange arrows represent the place where the neighbor tips are inserted. Scale bar = 1 µm.

Supplementary Table

Supplementary Table 1. Dimensions of the 45 coccoliths obtained from 3D-CXDI measurements.

Coccolith number	Species	Plate major axis(µm)	Plate minor axis (µm)	Grid major axis (µm)	Grid minor axis (µm)	Plate eccentricity	Grid eccentricity	Grid perimeter (µm)	Number of elements	Proximal rim length (µm)	Tube height (µm)	Masse (pg)
1	*R. parvula*	1,97	1,56	1,01	0,64	0,61	0,78	2,62	24	0,55	0,24	0,79
2	*R. parvula*	2,28	1,74	1,16	0,72	0,65	0,78	2,99	28	0,56	0,23	0,99
3	*R. parvula*	2,28	1,79	1,22	0,77	0,62	0,78	3,16	27	0,64	0,26	1,07
4	*R. parvula*	2,35	1,97	1,25	0,82	0,55	0,75	3,29	28	0,66	0,27	1,17
5	*R. parvula*	2,36	1,76	1,22	0,72	0,67	0,80	3,10	26	0,59	0,23	0,86
6	*R. parvula*	2,37	1,86	1,32	0,79	0,62	0,80	3,36	31	0,64	0,24	1,17
7	*R. parvula*	2,40	1,82	1,33	0,74	0,65	0,83	3,32	29	0,64	0,25	1,19
8	*G. ericsonii*	1,60	1,36	0,59	0,41	0,53	0,72	1,59	na	0,48	0,23	0,52
9	*G. ericsonii*	1,98	1,47	0,90	0,49	0,67	0,84	2,24	na	0,54	0,28	1,01
10	*G. ericsonii*	2,02	1,55	1,04	0,57	0,64	0,84	2,58	24	0,51	0,28	0,94
11	*G. ericsonii*	2,27	1,83	1,18	0,70	0,59	0,80	2,99	28	0,57	0,27	1,28
12	*G. ericsonii*	2,31	1,86	1,18	0,69	0,59	0,81	2,99	28	0,62	0,28	1,42
13	*E. huxleyi RCC1212*	2,87	2,39	1,14	0,74	0,56	0,76	2,99	27	0,89	0,51	1,53
14	*E. huxleyi RCC1212*	3,46	2,94	1,30	0,76	0,53	0,81	3,30	30	1,21	0,63	3,86
15	*E. huxleyi RCC1212*	3,65	3,23	1,41	0,84	0,47	0,80	3,58	31	1,18	0,57	3,59
16	*E. huxleyi RCC1212*	3,80	3,24	1,47	0,88	0,52	0,80	3,75	34	1,15	0,64	4,55
17	*E. huxleyi RCC1212*	4,41	3,86	1,64	1,06	0,48	0,76	4,29	38	1,50	0,65	5,10
18	*E. huxleyi RCC1216*	3,03	2,42	1,45	0,78	0,60	0,84	3,58	32	0,95	0,36	2,68
19	*E. huxleyi RCC1216*	3,06	2,39	1,45	0,86	0,62	0,80	3,69	33	0,81	0,39	2,46
20	*E. huxleyi RCC1216*	3,45	2,75	1,57	1,00	0,61	0,77	4,09	36	0,90	0,42	3,23
21	*E. huxleyi RCC1216*	3,47	2,72	1,54	0,99	0,62	0,76	4,01	33	0,94	0,46	3,23
22	*E. huxleyi RCC1216*	3,62	2,85	1,60	1,02	0,62	0,77	4,16	35	0,99	0,45	4,00
23	*E. huxleyi RCC1216*	3,63	2,93	1,59	1,12	0,59	0,71	4,29	37	0,90	0,45	3,54
24	*E. huxleyi P41*	3,14	2,56	na	na	0,58	na	3,81	34	na	0,50	5,04
25	*E. huxleyi P41*	3,14	2,55	na	na	0,58	na	3,81	34	na	0,44	4,55
26	*E. huxleyi P41*	3,48	2,95	na	na	0,53	na	3,59	32	na	0,47	6,57
27	*E. huxleyi P41*	3,54	2,94	na	na	0,56	na	4,15	37	na	0,51	7,54
28	*E. huxleyi P41*	3,54	2,94	na	na	0,56	na	4,15	37	na	0,51	7,54
29	*E. huxleyi P41*	3,58	2,90	na	na	0,59	na	3,92	35	na	0,49	7,44
30	*G. muellerae*	3,08	2,56	1,40	0,98	0,56	0,71	3,77	na	0,72	0,46	3,74
31	*G. muellerae*	3,27	2,67	1,45	0,86	0,58	0,81	3,69	na	0,75	0,46	4,03
32	*G. muellerae*	3,72	3,08	1,75	1,26	0,56	0,69	4,76	na	0,90	0,57	6,44
33	*G. muellerae*	3,76	3,38	1,56	1,18	0,44	0,66	4,32	na	1,01	0,54	7,55
34	*G. muellerae*	3,90	3,42	1,60	1,13	0,48	0,71	4,32	na	1,08	0,54	8,22
35	*G. muellerae*	3,90	3,26	1,83	1,33	0,55	0,69	4,99	na	0,91	0,53	6,82
36	*G. oceanica*	3,95	3,30	1,93	1,25	0,55	0,76	5,05	na	0,95	0,65	7,19
37	*G. oceanica*	4,00	3,23	1,81	1,19	0,59	0,75	4,76	na	0,90	0,66	7,50
38	*G. oceanica*	4,30	3,57	1,87	1,35	0,56	0,69	5,09	na	0,99	0,67	9,10
39	*G. oceanica*	4,31	3,72	1,63	1,05	0,51	0,76	4,26	na	0,93	0,64	7,82
40	*G. oceanica*	4,50	3,83	1,94	1,38	0,52	0,70	5,25	48	1,02	0,69	9,49
41	*G. oceanica*	4,67	4,02	2,00	1,43	0,51	0,70	5,42	na	1,05	0,74	11,28
42	*G. oceanica*	4,76	4,08	2,16	1,60	0,52	0,67	5,94	na	1,09	0,80	12,60
43	*G. oceanica*	4,80	4,07	2,20	1,56	0,53	0,71	5,95	na	1,11	0,81	14,10
44	*G. oceanica*	5,60	4,81	2,48	1,71	0,51	0,72	6,64	na	1,41	1,02	20,60
45	*G. oceanica*	5,87	5,06	2,58	1,79	0,51	0,72	6,92	61	1,40	1,04	23,05
